# Gait apraxia evaluation in normal pressure hydrocephalus using inertial sensors. Clinical correlates, ventriculoperitoneal shunt outcomes, and tap-test predictive capacity

**DOI:** 10.1186/s12987-022-00350-y

**Published:** 2022-06-23

**Authors:** Alberto Ferrari, David Milletti, Pierpaolo Palumbo, Giulia Giannini, Sabina Cevoli, Elena Magelli, Luca Albini-Riccioli, Paolo Mantovani, Pietro Cortelli, Lorenzo Chiari, Giorgio Palandri

**Affiliations:** 1 Science & Technology Park for Medicine, TPM, Mirandola, Modena Italy; 2 Department of Engineering “Enzo Ferrari”, University of Modena and Reggio Emilia, Modena, Italy; 3 Department of Electrical, Electronic, and Information Engineering “Guglielmo Marconi” (DEI), University of Bologna, Bologna, Italy; 4 Unit of Rehabilitation Medicine, IRCCS Istituto delle Scienze Neurologiche di Bologna, Bellaria Hospital Via Altura 3, 40139 Bologna, Italy; 5 Department of Biomedical and Neuromotor Sciences (DIBINEM), University of Bologna, Bologna, Italy; 6 Unit of Neurology, IRCCS Istituto delle Scienze Neurologiche di Bologna, Bologna, Italy; 7 Unit of Neuroradiology, IRCCS Istituto delle Scienze Neurologiche di Bologna, Bologna, Italy; 8 Unit of Neurosurgery, IRCCS Istituto delle Scienze Neurologiche di Bologna, Bologna, Italy; 9 Health Sciences and Technologies Interdepartmental Center for Industrial Research (CIRI-SDV), University of Bologna, Bologna, Italy

**Keywords:** Idiopathic normal pressure hydrocephalus, Gait analysis, Ventriculoperitoneal shunt, Tap test, Gait apraxia

## Abstract

**Background:**

Idiopathic normal pressure hydrocephalus (iNPH) is a neurological condition with gait apraxia signs from its early manifestation. Ventriculoperitoneal shunt (VPS) is a surgical procedure available for treatment. The Cerebrospinal fluid Tap Test (CSF-TT) is a quick test used as selection criterion for VPS treatment. Its predictive capacity for VPS outcomes is still *sub judice*. This study is aimed to test the hypothesis that wearable motion sensors provide valid measures to manage iNPH patients with gait apraxia.

**Methods:**

Forty-two participants of the Bologna PRO-Hydro observational cohort study were included in the analyses. The participants performed the Timed Up and Go (TUG) and the 18 m walking test (18mW) with inertial sensors at baseline, three days after the CSF-TT, and six months after VPS. 21 instrumental variables described gait and postural transitions from TUG and 18mW recordings. Furthermore, participants were clinically assessed with scales (clinical variables). We tested the hypothesis by analysing the concurrent validity of instrumental and clinical variables, their individual- and group-level responsiveness to VPS, and their predictive validity for VPS outcomes after CSF-TT.

**Results:**

The instrumental variables showed moderate to high correlation with the clinical variables. After VPS, most clinical and instrumental variables showed statistically significant improvements that reflect a reduction of apraxic features of gait. Most instrumental variables, but only one clinical variable (i.e., Tinetti POMA), had predictive value for VPS outcomes (significant adjusted R^2^ in the range 0.12–0.70).

**Conclusions:**

These results confirm that wearable inertial sensors may represent a valid tool to complement clinical evaluation for iNPH assessment and prognosis.

**Supplementary Information:**

The online version contains supplementary material available at 10.1186/s12987-022-00350-y.

## Background

Idiopathic normal pressure hydrocephalus (iNPH) is a syndrome characterised by normal cerebrospinal fluid (CSF) pressure associated with chronic ventricular dilation. Progressive gait impairments, urinary incontinence, and cognitive deficits define the clinical triad that highlights its onset [1]. Usually, gait alterations represent an early manifestation of the syndrome, accompanied by motor disabilities and increased fall risk, whereas cognitive impairment and urinary incontinence might appear in a later stage [2]. For this reason, the correct definition of gait alterations and their clinical and quantitative description, together with a differential diagnosis from similar motor conditions (iNPH mimics), represent critical challenges to address the syndrome in its early phase [3]. Motor alterations often appear as a complex syndrome, characterised by large base of support, reduced gait speed, stride length, foot clearance, left-to-right coordination, and difficulty in postural transitions, not depending on motor, sensory or cerebellar deficits [4]. For the first time in 1977 Miller-Fisher thoroughly described iNPH gait alterations using the term *"gait apraxia"* [5]. Considering that the classical definition of apraxia of gait is “*loss of ability to properly use lower limbs in the act of walking which cannot be accounted for by demonstrable sensory impairment or motor weakness”* [6], also today this term represents a broadly accepted definition of iNPH gait alterations in literature.

Once iNPH is adequately diagnosed, treating it with the insertion of a ventriculoperitoneal shunt (VPS) allows draining the CSF, which in turn can lead up to a partial or complete recovery [7]. On the other hand, delayed intervention can cause disease progression to a point where treatment may be no longer effective. Patient selection for VPS treatment is crucial to date, but clinical predictors for a successful outcome are poorly investigated and known [8].

As reported by a recent review and meta-analysis by Scully et al. [9], there is still a strong need for an accurate tool to predict shunt outcomes to facilitate standardization of care and optimal management of patients. The CSF-Tap Test (CSF-TT) is an assessment test used to predict iNPH patients with positive shunt-response [10]. It consists in removing 30–50 ml of CSF via lumbar puncture and clinically assessing the patient before and after the CSF removal to verify symptoms modifications [10]. The simplicity of this procedure allows it to be performed in an outpatient environment and an improvement in gait quality is considered to be predictive of a positive response to VPS surgery. However, evidence supporting its validity is still inadequate [11]. Moreover, the degree of change in motor symptoms to be considered a positive response has not yet been established [8, 12]. In a recent review, Mihalj et al. quantified the accuracy of CSF-TT in screening patients for shunting as 62%, but with a negative predictive value as low as 37% [13].

Visual observation is still the main procedure to assess motor performance. However, clinical tests and scales such as the timed up and go test (TUG), the Tinetti Performance Oriented Mobility Assessment (POMA), and the self-selected gait speed are considered beneficial ancillary evaluations when associated with CSF-TT [11]. In particular, Scully et al. [9] found a moderate prognostic value of TUG and 18-m walking test (18mW) gait speed in predicting shunt outcomes among iNPH patients. Still, these tests suffer particularly in terms of specificity, estimated at about 0.63 and 0.67 for TUG and 18mW respectively. To avoid underestimating VPS responders, Scully et al. also highlighted the importance of not using these tests in isolation [9].

In clinical practice, POMA is one of the most used scales and is considered a helpful tool when associated with a CSF-TT [14]. However, as reported in a systematic review by Gor-Garcia-Fogeda et al. [14], this type of assessment fails to capture objectively the possible diversities of motor manifestations and can result in subjective, inconsistent interpretations of the motor response. Convenient and accessible, yet highly accurate objective tools are therefore needed for patients’ assessment and management.

Instrumented gait analysis represents a promising paradigm for addressing this need by objectively assessing gait and balance improvements and unveiling details that are not easily accessible by simply using clinical observations or clinical scales [15–18]. The inertial measurement units (IMUs) are increasingly employed as motion sensors in instrumenting motor tests in the clinical routine [19]. Nowadays, IMUs are wireless, lightweight, cost-effective, and operate on commercially available mobile devices, thus allowing the plug-and-play execution of gait analysis tests without the need for specialized personnel or dedicated laboratories [20, 21].

Only a few studies employed an instrumented gait analysis for studying iNPH gait apraxia and its improvement after CSF-TT [17, 21–27]. Stolze et al. reported gait speed and stride length as the most responsive parameters in a pre vs. 24 h post-CSF-TT comparison [22]. A few years later, Williams et al. confirmed an improvement in gait velocity and described a reduction of double support time and cadence [26]. Recently, Allali et al. found a significant improvement of gait speed in single and dual tasking during the 10 m walk test in a pre vs. post-CSF-TT comparison [23]. Colella et al. [24] confirmed the stride length to be one of the most reliable parameters in evaluating iNPH’s gait performance. In a pre vs. 24 h post-CSF-TT study, Lim et al. reported significant improvements of gait velocity, stride length, and step width and decreases in stride time and stride length variability [27]. Other studies focused on TUG parameters. Bovonsunthonchai et al. compared performances on TUG pre vs. post-CSF-TT, revealing a significant improvement in the sit-to-stand transition, walking time, and the number of steps employed to turn [25]. Ferrari et al. [17] reported a reduction of double support duration and an increase of cadence from baseline to 72 h post-CSF-TT in a 18mW and TUG, and an increase of stride length in the 18mW.

On iNPH patients instrumented with inertial sensors, He et al. found a decreased cadence, reduced gait speed, a higher duration of double support, decreased elevation at mid-swing, reduced foot strike angle, shorter stride length, difficulty in turning, and impaired balance functions [21]. They also showed that most previously listed gait manifestations were significantly improved after external lumbar drainage in responders to external lumbar drainage.

## Methods

### Aim

To the best of our knowledge, no study used inertial sensors for measuring patients' response to VPS and evaluate the CSF-TT predictive capacity on VPS outcome. More so, instrumental parameters derived from IMUs signals recorded during functional tests (e.g., TUG and walking tests) have not been studied for their associations with clinical scales commonly used for iNPH assessment. Knowing these clinical correlates would be essential for establishing concurrent validity and their utility in clinical routine. Therefore, the ability of IMUs to provide valid measures to manage iNPH patients with gait apraxia is still a hypothesis. The aim of the present study was to test this hypothesis, evaluating instrumental variables derived from IMUs during TUG and 18mW. In particular, we aim to test the validity of instrumental variables on three aspects, namely (i) their concurrent validity, (ii) their responsiveness to VPS, and iii) their ability in CSF-TT to predict VPS outcomes. Clinical scales traditionally employed with iNPH patients have been used as comparators.

### Participants

Data collected in this study are part of the Bologna PRO-Hydro study, an observational cohort study conducted in the IRCCS Istituto delle Scienze Neurologiche di Bologna between May 2015 and November 2019 [16]. Inclusion and exclusion criteria for the population eligible for the PRO-Hydro study, thoroughly described in Giannini et al. [16], in summary, are: (i) aged over 50 years old, (ii) with suspicion of iNPH by clinical history and neuroimaging (CT or MRI scan), (iii) able to give verbal and written informed consent, and not presenting: i) severe psychiatric diseases or physical illness, ii) addiction to drugs, or iii) a clinical history possibly causing ventricular dilation. Within the PRO-Hydro population, patients who received VPS and could perform the clinical and instrumental tests were enrolled in this study. The analyses of this study were conducted after having collected data on 42 eligible participants in the PRO-Hydro study. This sample size provides power to detect at least a moderate effect size (power = 80%, correlation coefficient = 0.42, alpha = 0.05) according to Cohen et al. [28].

This study was approved by the local ethics committee of the health service of Bologna (Cod. CE: 14131, 23/02/2015), and was conducted in agreement with principles of good clinical practice. All participants gave their written informed consent to participate according to the Declaration of Helsinki.

### Protocol

The protocol for patients’ diagnosis and treatment has been detailed in Giannini et al. [16]. Patients with a suspicion of iNPH underwent a 3T MRI Brain Scan and an outpatient assessment. The diagnosis was assigned upon consensus by a multidisciplinary team (composed of neurologists, neurosurgeons, neuroradiologists, and physiatrists) after reviewing all baseline clinical and neuropsychological information and the results from blood and CSF tests. Based on the diagnosis and taking into consideration comorbidities and vascular risk factors, eligible patients for VPS were identified and invited for surgical procedure.

Patients’ potential response to VPS was evaluated with CSF-TT by removing 30 ml of CSF using a 20-gauge spinal needle in a lateral supine position.

Clinical and instrumental evaluations were repeated before the CSF-TT (preTT) and, as proposed by Ferrari et al. [17], 72 h after the CSF-TT (postTT). Shunt surgery consisted of the implantation of programmable valves with an antisiphon device through the right frontal burr hole. The median valve’s opening pressure was 120 mmH_2_0 [16].

Patients were clinically and instrumentally evaluated once again six months after the VPS (postVPS).

### Measurements

Clinical and instrumental measurements used for the aims of this study are listed in Table 1. Balance and motor abilities were assessed with (POMA) [29], Gait Status Scale (GSS) [30], number of steps to make a 180° turn (nSteps2turn) [16], TUG, and 18mW [31]. The severity of the disease on the three symptoms of the triad (gait, cognition, and urinary disturbances) was assessed with iNPH Grading Scale (iNPH-GS) [30]. These measurements were executed preTT, postTT, and postVPS. Finally, the level of disability was assessed with Functional Independence Measure (FIM) [32] and Rankin Scale [33] preTT and postVPS.

**Table 1:** Name and description of clinical and instrumental measurements

Variable (unit of measurement)	Description
Clinical variables	
nSteps2turn	Number of steps for 180° turn
Tinetti Balance	Tinetti balance score. Range 0–16, positively oriented [29]
Tinetti Gait	Tinetti gait score. Range 0–12, positively oriented [29]
Tinetti Total	Tinetti total score = Tinetti balance score + Tinetti gait score. Range 0–28, positively oriented. The Tinetti scale is also known as Performance Oriented Mobility Assessment (POMA) [29]
GSS	Gait Status Scale. Scale for evaluating postural and gait disturbances in iNPH patients. Eight items, range 0–16, negatively oriented [30]
iNPH-GS	iNPH Grading Scale. Scale to grade the severity of patients with iNPH on three domains: cognition, gait, and urinary disturbances. Range 0–12, negatively oriented [30]
FIM	Functional Independence Measure. It measures the level of disability in patients with functional mobility impairments. Range 18–126, positively oriented [32]
Rankin Scale	Scale for measuring disability. Often used for stroke or other neurological patients. Range 0–6, negatively oriented [33]
TUG—18mW	
TestDuration (s)	Time spent to complete the test
TotalSteps (steps)	Total number of steps to complete the test
WalkTime [TUG] (s)	Time spent walking during the TUG
StandTime [TUG] (s)	Time to stand up from the chair
SitTime [TUG] (s)	Time to sit down on the chair
TurnSteps [TUG]	Number of steps for turning 180° around the cone during TUG execution
Cadence (steps/min)	Mean number of steps per minute
StrideLength (cm)	Mean value of strides length
DoubleSupport (% of gait cycle)	Mean value of the percentage of gait cycles spent in double support phase (both feet touching the ground)
GaitSpeed (cm/s)	Mean of the gait speed
TrunkInclination (degrees)	Mean of the trunk inclination on the sagittal plane with respect to the vertical axis, determined according to [17]
maxTC1 (cm)	Maximum foot clearance occurring in the time frame between foot off and mid swing events, determined according to [42]
maxTC2 (cm)	Maximum foot clearance between mid swing and initial contact, determined according to [42]
minTC (cm)	Minimum foot clearance between mid swing and initial contact, determined according to [42]
PitchAtTC2 (degrees)	Angle of foot inclination in the sagittal plane in correspondence of the maximum foot clearance between the phase of mid swing and initial contact. This value is obtained after the removal of inclination offset due to the sensor position on the shoe (the minus sign of the angle is therefore misleading)
pci	Phase coordination index, expressing variability and inaccuracy in gait bilateral coordination as proposed by Plotnik et al. [43]
StrideSD (cs)	Standard deviation of stride time, expressing gait variability
psdF (Hz)	Parameter indicative of the frequency at which the energy of feet motion is maximum. The medio-lateral angular velocity of each foot is detrended and their power spectral densities are then calculated with the Welch method. The two frequencies where these—right and left—power spectral densities are maximum are determined. psdF is the mean between the two frequencies
psdW (Hz)	Parameter indicative of the energy dispersion of feet motion. The medio-lateral angular velocity from each foot is detrended and their power spectral densities are calculated with the Welch method. The—right and left—widths of the base of the peaks of these two power spectral densities are determined. psdW is the mean between the two widths

The two motor tests to assess functional balance and mobility were 18mW [9, 31] and TUG [16]. During 18mW, patients were instructed to walk straight at a self-selected pace along a large and empty 30 m long corridor. During TUG, patients raised from a chair with armrests, walked 3 m forward, turned 180° around a traffic cone, walked 3 m backward, and sat back on the same chair. Both tests were repeated three times to increase reliability and filter out lack of attention and habituation effects.

**Fig. 1:**
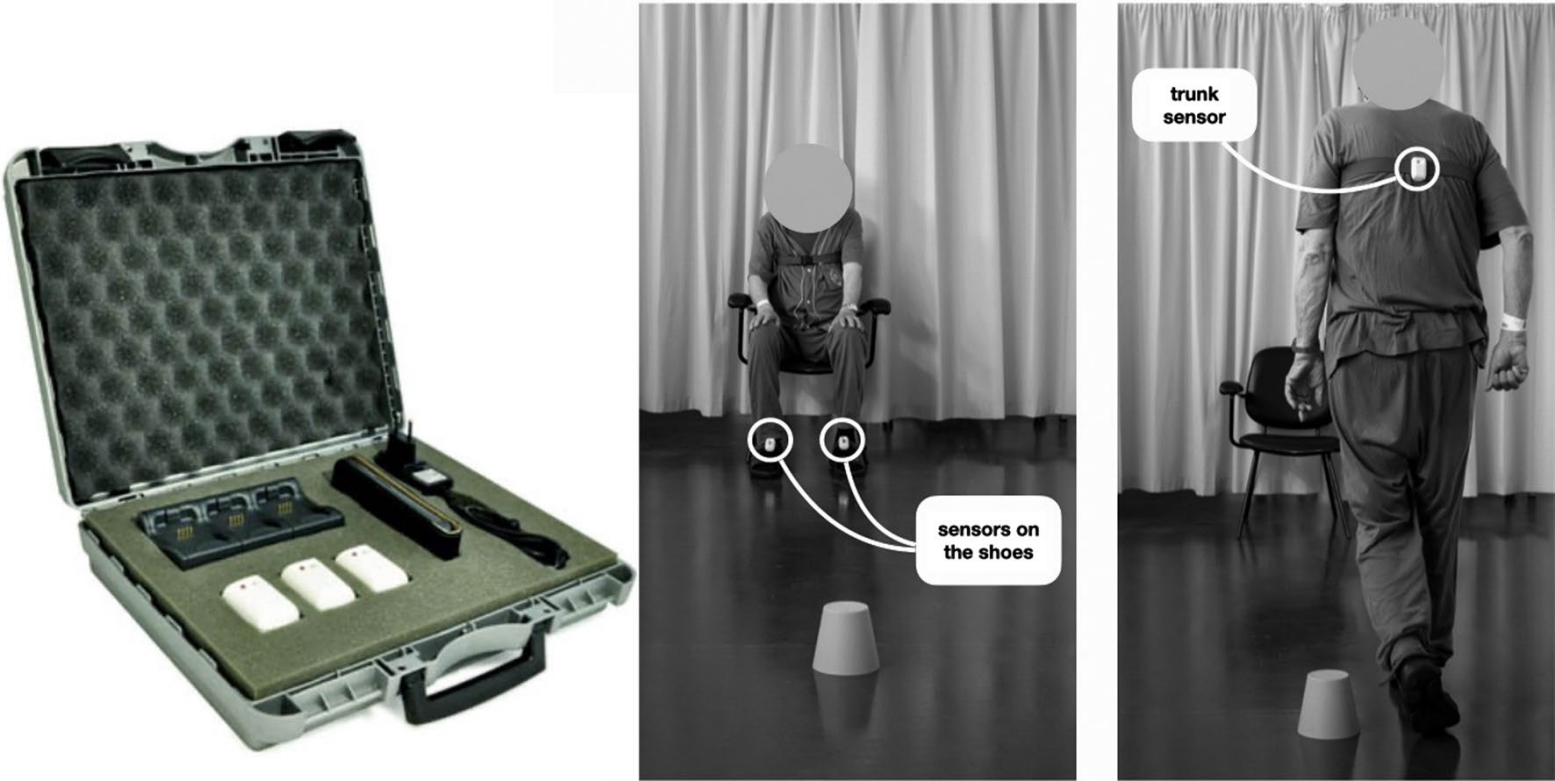
mGait system (mHealth Technologies, Italy). Three inertial measurement units (on the left) are attached two on top of the shoes and one over the trunk

The 18mW and the TUG were instrumented using three inertial sensors: two worn on shoes and one on trunk (mGAIT, mHealth Technologies, Italy; see Fig. 1, [see Additional file 1]) [17]. In particular, IMUs were connected via Bluetooth to an Android smartphone using an app that implemented ad-hoc algorithms to detect gait cycle events in real real-time and calculate gait spatio-temporal parameters [20, 34]. In addition, trunk acceleration signals recorded during TUG were processed offline using Matlab 2019b (Mathworks Inc., USA) to calculate the sit-to-stand duration (standTime), the stand-to-sit duration (sitTime), and the time spent in walking (walkTime) [17].

### Statistical analyses

Concurrent validity of the instrumental variables and the clinical scales was tested calculating the Spearman’s rank correlation coefficients between these two sets of variables at preTT. Repeated measures of instrumental variables at preTT were averaged.

The individual-level responsiveness of the instrumental variables was evaluated calculating their minimal detectable change [35]. The minimal detectable change with 95% confidence level (MDC) was derived from the intraclass correlation coefficient (ICC 2,k [36]) from preTT repeated measurements [37]. The group-level responsiveness of the instrumental variables to VPS was evaluated calculating their change between preTT and postVPS with repeated-measures analysis of variance (type II Wald chi-squared test). In particular, for each instrumental variable, we fitted a linear mixed-effect model with the instrumental variable modelled as the dependent variable, the time modelled as a fixed effect, and the correlation between patient-specific repeated measures modelled with a random intercept [38]. The change on clinical scales between preTT and postVPS was evaluated with the Wilcoxon-Mann–Whitney test.

To determine the prognostic value of CSF-TT for VPS outcomes, for each clinical or instrumental variable, a likelihood ratio test was performed between two linear nested models, namely a base and a wide model [39]. In the base model, the value of the variable at postVPS was modelled as the outcome variable, explained by its value at preTT. The wide model was conceived as the base model but with the addition of one regressor being the difference between the performances at preTT and postTT (Δ_postTT-preTT_). Repeated measures of instrumental variables at each time point (preTT, postTT, postVPS) were averaged. The goodness of fit of the models was evaluated with the adjusted R^2^ statistics.

All the statistical analyses were run using the R statistical software (https://www.r-project.org/).

## Results

**Fig. 2:**
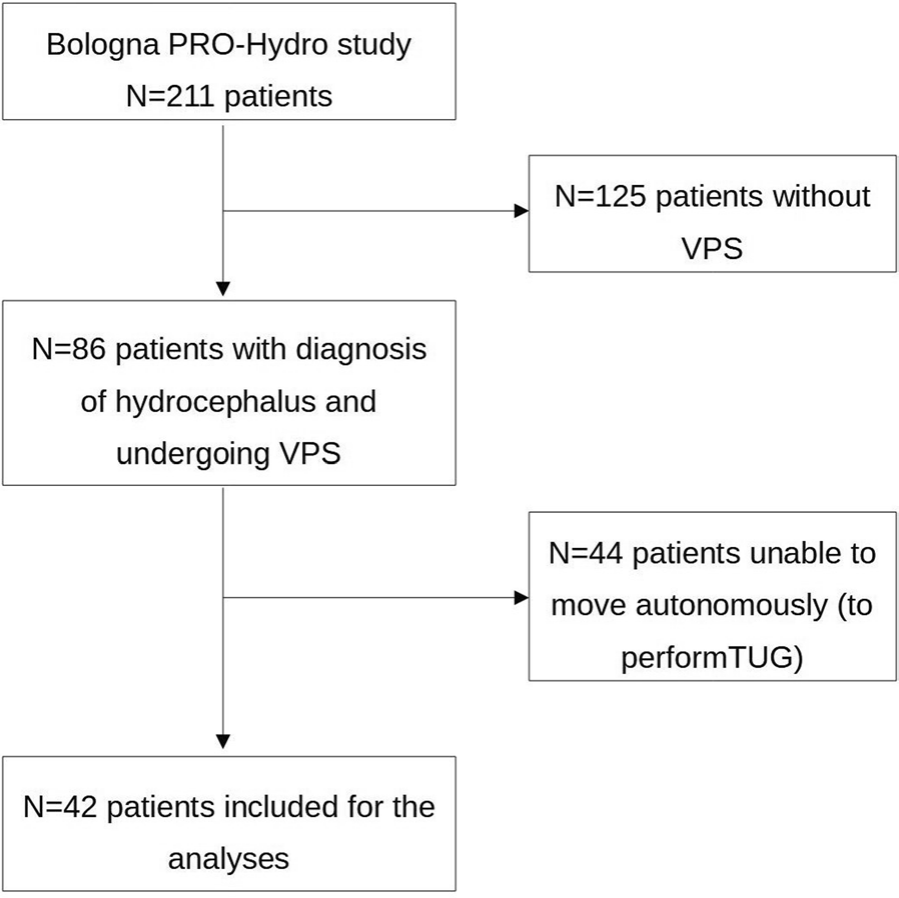
Flowchart of patients’ selection for inclusion in the analyses. Out of the 42 patients included for the analyses, 3 had no complete sensor data on the 18mW

Eighty-six patients included in the Bologna PRO-Hydro study between May 2015 and November 2019 were diagnosed with iNPH and underwent the VPS. Among these, 42 patients who were able to move independently and had completed the instrumental TUG acquisition at preTT, postTT, and postVPS, were considered for the analyses of this study (Fig. 2). Mean (± standard deviation, SD) age was 75.2 ± 4.0 years. Most patients were women (n = 27, 64%), with a body mass index of 26.9 ± 4.0 kg/m^2^. At baseline, median iNPH-GS and Tinetti POMA were respectively 6 (interquartile range, IQR 4.75–7) and 20 (IQR 18–23). The median Rankin Scale was 2 (IQR 1–3) (Table 2). The mean number of days between preTT and postTT was 4.4, between preTT and VPS was 121, and bewteeen VPS and postVPS was 166.8.

**Table 2:** Descriptive statistics of the patients included for the analyses at baseline

N	42	N with missing data
Age (years)	75.2 ± 4.0	1
Sex (male)	15 (36%)	0
BMI (kg/m.^2^)	26.9 ± 4.0	1
INPH-GS	6 (IQR 4.75–7)	2
Tinetti total	20 (IQR 18–23)	1
Rankin Scale	2 (IQR 1–3)	1

Values are given as mean ± standard deviation, number and frequency (%), or median and interquartile range (IQR)

BMI body mass index

**Fig. 3:**
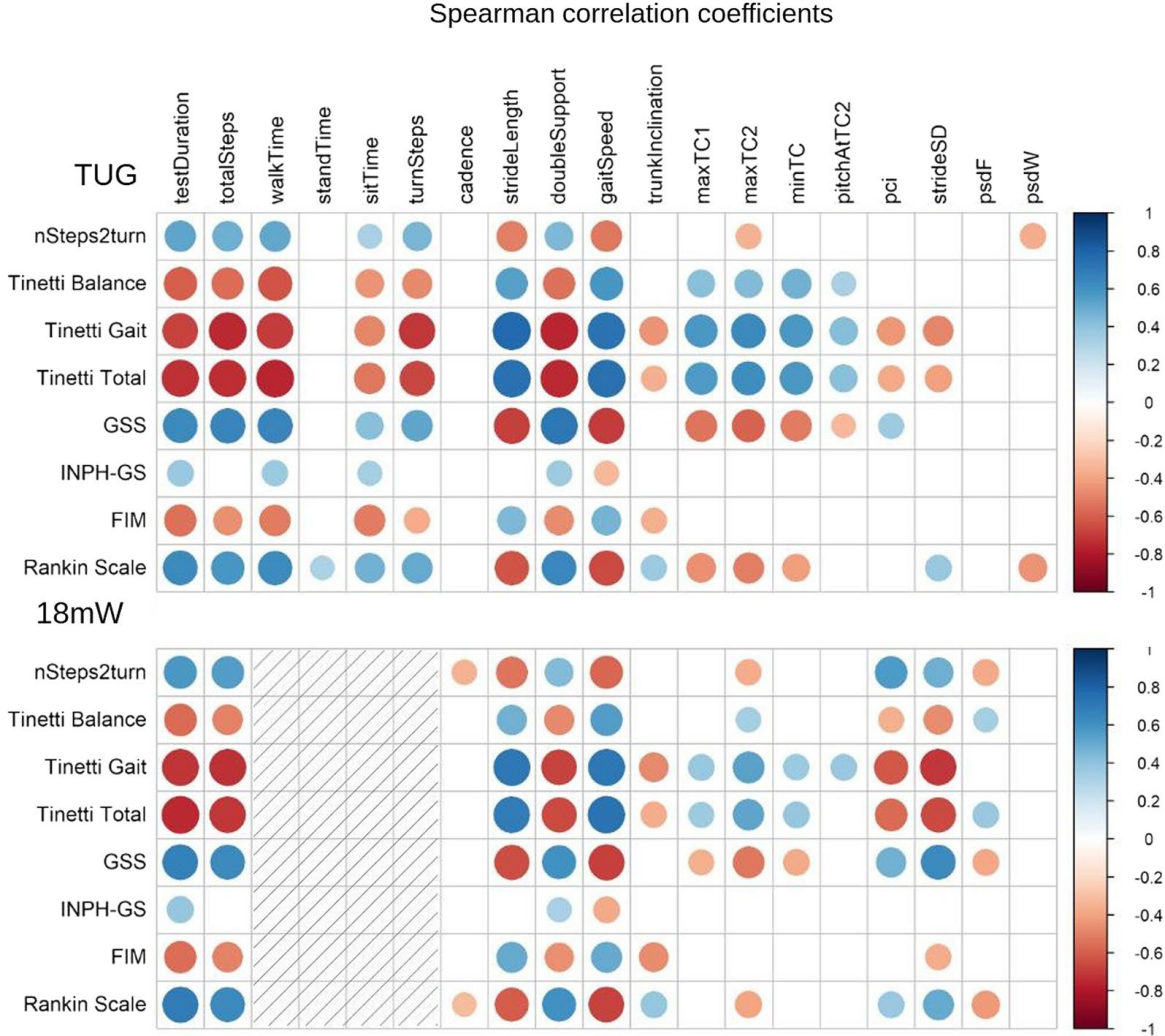
Spearman’s correlation coefficients between clinical and instrumental variables. Variables measured preTT. Only statistically significant coefficients are shown (p < 0.05)

Most instrumental gait parameters were significantly correlated with clinical scores (Fig. 3, [see Additional file 1: Table S1]). Correlation coefficients were in the expected direction of the association. Only FIM, INPH-GS and nSteps2turn did not show high correlation coefficients with any instrumental variables, with values ranging from 0.33 to 0.58. Tinetti Balance, Tinetti Gait, Tinetti total, GSS and Rankin scale obtained statistically significant coefficients in the range 0.32–0.77, with at least one instrumental parameter showing a coefficient with an absolute value above 0.6. Five TUG variables (testDuration, walkTime, sitTime, doubleSupport, gaitSpeed) and three 18mW variables (testDuration, doubleSupport, gaitSpeed) correlated with all clinical parameters.

**Fig. 4:**
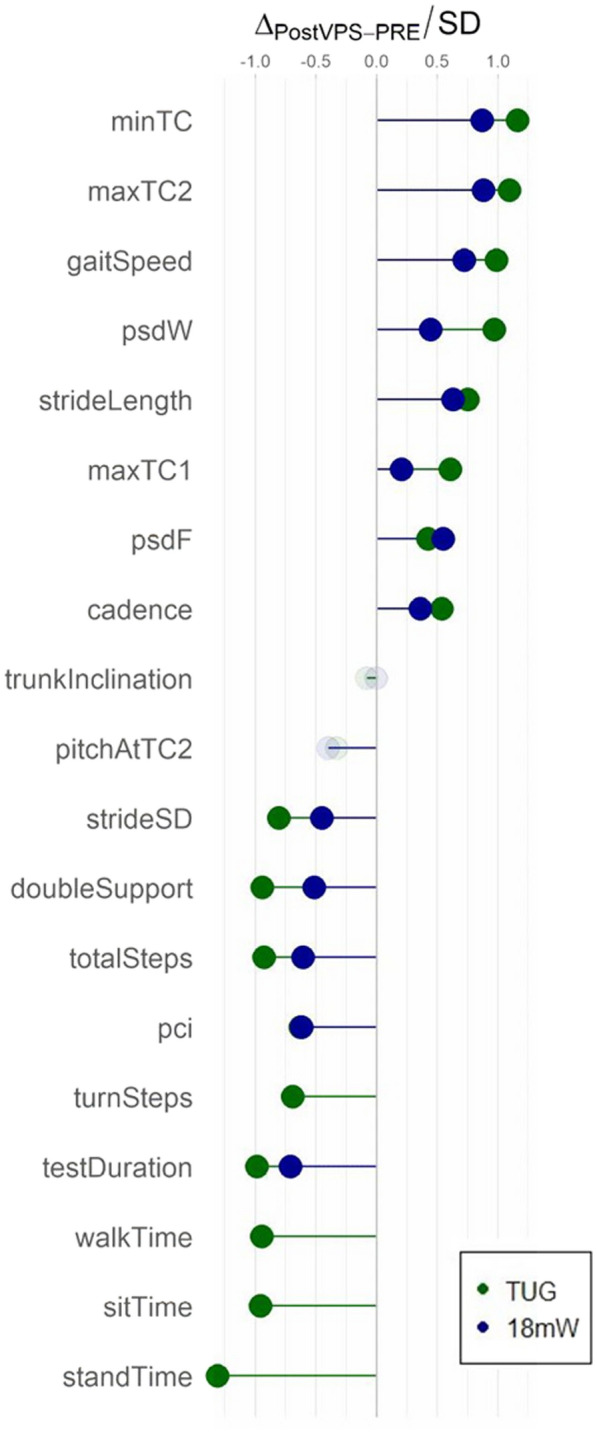
Relative variations of instrumental variables between preTT and postVPS. Variations are normalized to the inter-subject standard deviation (SD) of each variable (Cohen’s effect size). Green = TUG, blue = 18mW. Non-significant changes are shown with pale colours

Most instrumental variables showed excellent reliability (ICC above 0.90, [see Additional file 1: Table S2]). Besides, MDC testDuration, totalSteps, strideLength, doubleSupport, gaitSpeed and minTC were smaller than the mean group-level change after VPS. All clinical evaluations except FIM significantly improved between preTT and postVPS. In particular, Tinetti POMA score improved of 4.5 points (95% confidence interval (C.I.) [3.5, 5.5], median preTT: 20, postVPS: 24), while GSS improved of 2.5 points (95% C.I. [− 3.5, − 1.5], median preTT: 5, postVPS: 2). All instrumental variables significantly changed between preTT and postVPS, except trunkInclination and pitchAtTC2 measured in TUG and 18mWT. TUG testDuration decrease was − 7.66 s (95% C.I. [− 9.47, − 6.09] s, mean preTT: 24.07 s, postVPS: 16.41 s), while 18mW testDuration decrease was -8.1 s (95% C.I. [− 10.62, − 5.73] s, mean preTT: 33.85 s, postVPS: 25.76 s; [see Additional file 1: Table S2 ]). Figure 4 reports the relative changes of instrumental variables after VPS, measured as the difference between preTT and postVPS divided by the inter-subject standard deviation (Cohen’s effect size). Such effect sizes range, in module, between 0.21 and 1.39.

**Fig. 5:**
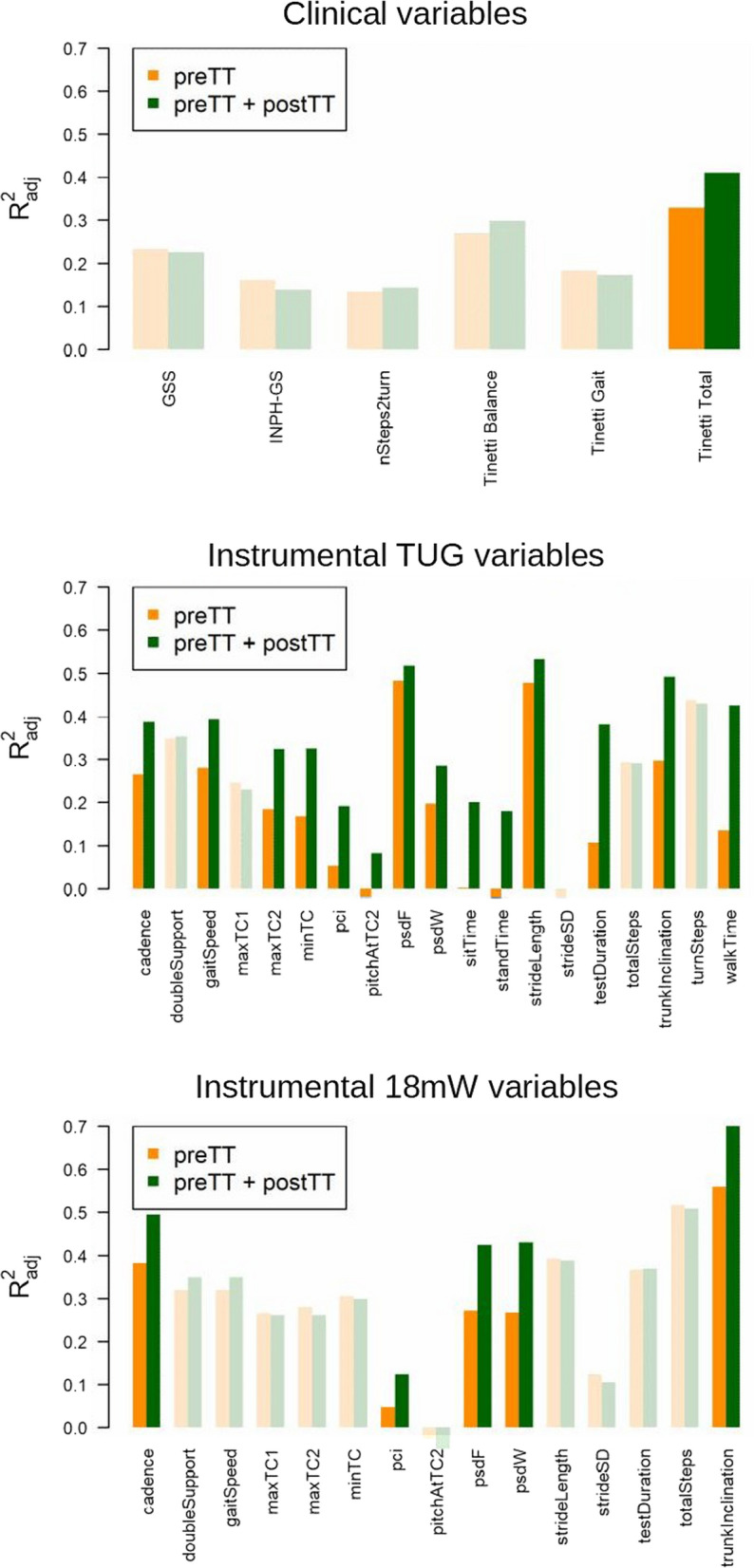
CSF-TT predictive capacity. Barplots for the accuracy (R^2^_adj_) of predicting postVPS clinical or instrumental variables, using their values at preTT or both at preTT and postTT. Variables for which postTT values do not lead to a significantly improved postVPS prediction are shown with pale colours

Table 3 and Fig. 5 show the results on the added prognostic value of the CSF-TT for predicting the clinical and instrumental outcomes postVPS. Among clinical variables, the Tinetti POMA Total score change after the CSF-TT had significant prognostic capacity for its postVPS value (p = 0.013). The Tinetti POMA score postVPS was predicted from its preTT value and its change after CSF-TT with an adjusted R^2^ of 0.41. All other clinical variables after CSF-TT did not show prognostic capacity for their values postVPS, with adjusted R^2^ of the prediction models ranging from 0.14 to 0.30.

**Table 3:** Models for predicting each feature postVPS (after 6 months), given their value at preTT (baseline), or both their value at preTT and their change after the TT (postTT)

	postVPS ~ preTT	postVPS ~ preTT + Δ_postTT-preTT_	Likelihood ratio test	
	R^2^_adj_	R^2^_adj_	χ^2^	p-value
Clinical				
nSteps2turn	0.13	0.14	1.409	0.235
Tinetti Balance	0.27	0.30	2.661	0.103
Tinetti Gait	0.18	0.17	0.529	0.467
Tinetti Total	0.33	0.41	6.213	**0.013**
GSS	0.23	0.23	0.637	0.425
iNPH-GS	0.16	0.14	0.083	0.773
TUG				
TestDuration	0.11	0.38	16.437	**0.0001**
TotalSteps	0.29	0.29	0.914	0.339
WalkTime	0.13	0.42	18.151	**0.00002**
StandTime	-0.02	0.18	10.333	**0.001**
SitTime	0.00	0.20	10.392	**0.001**
TurnSteps	0.44	0.43	0.50	0.480
Cadence	0.26	0.39	8.714	**0.003**
StrideLength	0.48	0.53	5.715	**0.017**
DoubleSupport	0.35	0.35	1.386	0.239
GaitSpeed	0.28	0.39	8.163	**0.004**
TrunkInclination	0.30	0.49	14.693	**0.0001**
maxTC1	0.25	0.23	0.150	0.699
maxTC2	0.18	0.32	8.996	**0.003**
minTC	0.17	0.32	9.815	**0.002**
PitchAtTC2	-0.02	0.08	5.465	**0.019**
pci	0.05	0.19	7.338	**0.007**
StrideSD	-0.02	0.00	2.016	0.156
psdF	0.48	0.52	3.929	**0.047**
psdW	0.20	0.28	5.914	**0.015**
18mW				
TestDuration	0.37	0.37	1.214	0.271
TotalSteps	0.52	0.51	0.369	0.544
Cadence	0.38	0.49	8.882	**0.003**
StrideLength	0.39	0.39	0.792	0.374
DoubleSupport	0.32	0.35	2.761	0.097
GaitSpeed	0.32	0.35	2.761	0.097
TrunkInclination	0.56	0.70	16.055	**0.0001**
maxTC1	0.27	0.26	0.811	0.368
maxTC2	0.28	0.26	0.016	0.898
minTC	0.31	0.30	0.667	0.414
PitchAtTC2	− 0.03	− 0.05	0.153	0.695
pci	0.05	0.12	4.311	**0.038**
StrideSD	0.12	0.11	0.281	0.596
psdF	0.27	0.42	10.174	**0.001**
psdW	0.27	0.43	10.901	**0.001**

R^2^_adj_ indicates the model goodness of fit. Likelihood ratio tests between nested models indicate the added prognostic value of CSF-TT information. Significant p-values (< 0.05) are highlighted in bold

Among TUG instrumental variables, CSF-TT prognostic capacity was significant for testDuration, walkTime, standTime, sitTime, cadence, strideLength, gaitSpeed, trunkInclination, maxTC2, minTC, pitchAtTC2, pci, psdF, and psdW. The highest predictive accuracy was obtained for strideLength (adjusted R^2^ = 0.53). The strongest evidence for the added prognostic value of CSF-TT was obtained for walkTime (χ^2^ = 18.15, p = 2∙10^–5^).

Among 18mW variables, CSF-TT prognostic capacity was significant for cadence, trunkInclination, pci, psdF, and psdW. The highest predictive accuracy and evidence for the added prognostic value of CSF-TT were obtained for trunkInclination (adjusted R^2^ = 0.70, χ^2^ = 16.055, p = 10^–4^).

## Discussion

Idiopathic normal pressure hydrocephalus is a complex neurological syndrome with gait apraxia manifestations. To the best of our knowledge, this is the first observational cohort study able to combine objective instrumental measures from wearable motion sensors with the major clinical iNPH scales. We aimed to test the hypothesis that instrumental measures provide valid tool for iNPH management, including the evaluation of gait apraxia symptoms, their improvements after VPS, and prediction of VPS outcomes by means of CSF-TT.

We employed clinical and instrumental measurements for gait assessment on 42 patients at baseline (preTT), 72 h after the CSF-TT (postTT), and six months after VPS (postVPS).

At baseline, we found significant correlations among some clinical and instrumental variables, that indicate concurrent validity. Tinetti Balance, Tinetti Gait, Tinetti total, GSS, and Rankin Scale showed strong correlation with at least one instrumental variable. In particular, TUG variables (except StandTime) strongly correlated with Tinetti Gait and Total scores. Variables related to gait coordination (pci), variability (strideSD), and frequency (psdF) showed higher correlations with clinical variables when computed on 18mW than TUG, possibly due to the shorter path in TUG.

Both in TUG and 18mW cadence showed weak (0.32) or non-significant correlations with clinical variables. Thus, cadence is an abnormal feature of iNPH gait that improves post CSF-TT [17, 21, 26] but is not captured by clinical variables. As expected, most instrumental variables (in particular gait speed, both in TUG and 18mW) were correlated with the number of steps to make a turn (nSteps2turn). An increased nSteps2Turn is usually associated with a reduced ability to dissociate the motor program among head, trunk, and lower limbs (“enbloc” movement). This clinical feature typically represents a pathological sign of turning instability [40].

FIM and INPH-GS were the two clinical variables showing the weakest correlations with instrumental variables, probably due to their multidimensional nature with different non-motor domains.

Both instrumental and clinical variables (except trunkInclination, pitchAtTC2, and FIM) showed an improvement six months after VPS, suggesting a reduction of motor impairment. In particular, the decreased double support (doubleSupport), the increased foot clearance (maxTC1 and maxTC2), and the improved gait bilateral coordination (pci), are consistent with the idea of a reduction of shuffling gait. Interestingly, some TUG variables (standTime, sitTime) can instead reflect a favorable modification in apraxia of postural transitions [4]. The MDC for most instrumental variables were smaller than the mean group-level change after VPS. This demonstrates how instrumental variables can possibly be used in clinical practice to sensibly measure VPS improvements (see Additional file 1: Table S2).

Among clinical variables, FIM did not show a statistically significant improvement after VPS. Tinetti POMA significantly improved, with a median value of 4.5 points [95% C.I. 3.5–5.5]. This value is slightly inferior to the MDC at the individual level that is of at least 5 points [41]. GSS, Rankin scale, iNPH-GS and the number of steps to turn also improved six months after VPS.

This study also revealed a predictive capacity of the CSF-TT for VPS outcomes. Using motion sensors for CSF-TT evaluation enables to infer VPS outcomes on many different aspects of gait apraxia [15]. The modifications of many instrumental variables after the CSF-TT proved to be informative for predicting gait apraxia improvements six months after surgery. Trunk flexion reduction after CSF-TT demonstrated good predictive accuracy for VPS outcome although its improvement after CSF-TT was not statistically significant. On the other hand, among clinical variables, only the Tinetti POMA demonstrated prognostic capacity to infer VPS response.

## Limitations

Our study has some limitations. First, the exclusion from the studied population of 44 patients characterized by very severe gait disturbances, abasia or bed-ridden which could not complete motor tests (Fig. 1) limit the external validity of the study. Secondly, for feasibility reasons the Bologna PRO-Hydro study is affected by a variability in the time that elapses between the inpatient program and the VPS that goes from one week to two months [16]. Finally, despite its good predictive accuracy for VPS outcome, trunk flexion reduction after CSF-TT did not demonstrate a statistically significant improvement. Further research must be carried out to confirm this result.

## Conclusions

The results of this study support the hypothesis that the instrumental analysis of multiple spatiotemporal parameters of gait, derived from wearable inertial sensors applied to TUG and 18mW, can capture important clinical correlates and describe motor apraxia modification of iNPH patients six months after VPS. Interestingly, instrumental variables resulted sensible enough for measuring improvements of motor apraxia after VPS on individual patients thus can possibly be introduced in clinical practice.

Besides an overall correlation between instrumental variables and motor clinical scales, instrumented TUG and 18mW showed to be able to broaden the spectrum of analysis of iNPH gait by allowing a quantitative measure of those features contributing to overall motor performance. In particular, instead of considering only the total time and/or the total number of steps (possibly affected directly by cognitive impairments), the application of inertial sensors to the TUG and 18mW could expand the range of clinical assessment to parameters potentially less influenced by individual variability (for instance, TUG total time can be). Among clinical scales measured before and after CSF-TT, only the Tinetti POMA demonstrated a predictive capacity for VPS outcomes, whereas instrumented TUG and 18mW showed a predictive capacity for multiple spatiotemporal parameters. The findings of this study suggest that an evaluation protocol implementing the analysis of gait instrumental parameters together with clinical examinations and scales (TUG, 18mWT, Tinetti POMA, Rankin, iNPH-GS, GSS), represents a powerful tool in the management of iNPH patients affected by gait apraxia.

## Supplementary Information

**Additional file 1: Table S1.** Spearman’ rank correlation coefficients between clinical and instrumental variables. Only statistically significant coefficients are shown (p < 0.05). Variables measured preTT. **Table S2.** Clinical and instrumental variables results relative to TUG and 18mW. Clinical variables are summarised with their median preTT and postVPS; Δ_postVPS-preTT_ indicates the location parameter estimated by the Wilcoxon-Mann–Whitney test. Instrumental variables are summarised with the mean preTT and postVPS; Δ_postVPS-preTT_ indicates their difference; estimations are made with linear mixed-effect models. Relative reliability (ICC), absolute reliability (SEM), and individual-level responsiveness (MDC) of instrumental variables are also reported. *ICC* intraclass correlation coefficient, *SEM* standard error of measurement, *MDC* minimal detectable change. The variables whose MDC is lower than the Δ_postVPS-preTT_ (95% CI) are in bold.

## Data Availability

The data are available from the authors upon reasonable request.

## Abbreviations

iNPH: Idiopathic normal pressure hydrocephalus
CSF: Cerebrospinal fluid
VPS: Ventriculoperitoneal shunt
CSF-TT: Cerebrospinal fluid Tap Test
TUG: Timed up and go test
POMA: Tinetti Performance Oriented Mobility Assessment
18mW: 18-Meter walking test
IMUs: Inertial measurement units
postTT: 72 Hours after the cerebrospinal fluid Tap Test
preTT: Before the cerebrospinal fluid Tap Test
postVPS: Six months after the ventriculoperitoneal shunt
GSS: Gait Status Scale
nSteps2turn: Number of steps to make a 180° turn
iNPH-GS: Idiopathic normal pressure hydrocephalus Grading Scale
FIM: Functional Independence Measure
Δ_postTT__-preTT_: Difference between the performances at preTT and postTT
IQR: Interquartile range
C.I.: Confidence interval
