# Supplementary material for: Gait apraxia evaluation in normal pressure hydrocephalus using inertial sensors. Clinical correlates, ventriculoperitoneal shunt outcomes, and tap-test predictive capacity
Source: Fluids Barriers CNS. 2022 Jun 23;19:51. doi: 10.1186/s12987-022-00350-y (PMC9219204; doi:10.1186/s12987-022-00350-y)
Supplement: Supplementary file 1 — Additional file 1: Table S1. Spearman’ rank correlation coefficients between clinical and instrumental variables. Only statistically significant coefficients are shown (p < 0.05). Variables measured preTT. Table S2. Clinical and instrumental variables results relative to TUG and 18mW. Clinical variables are summarised with their median preTT and postVPS; ΔpostVPS-preTT indicates the location parameter estimated by the Wilcoxon-Mann–Whitney test. Instrumental variables are summarised with the mean preTT and postVPS; ΔpostVPS-preTT indicates their difference; estimations are made with linear mixed-effect models. Relative reliability (ICC), absolute reliability (SEM), and individual-level responsiveness (MDC) of instrumental variables are also reported. ICC intraclass correlation coefficient, SEM standard error of measurement, MDC minimal detectable change. The variables whose MDC is lower than the ΔpostVPS-preTT (95% CI) are in bold. [file 12987_2022_350_MOESM1_ESM.docx]

Gait apraxia evaluation in normal pressure hydrocephalus using inertial sensors. Clinical correlates, ventriculoperitoneal shunt outcomes, and tap-test predictive capacity.

# Alberto Ferrari, David Milletti, Pierpaolo Palumbo, Giulia Giannini, Sabina Cevoli, Elena Magelli, Luca Albini-Riccioli, Paolo Mantovani, Pietro Cortelli, Lorenzo Chiari, Giorgio Palandri

Supplemental material

**Table S1.** Spearman’ rank correlation coefficients between clinical and instrumental variables. Only statistically significant coefficients are shown (p<0.05). Variables measured preTT.

|  | nSteps2turn | iNPH-GS | Tinetti Balance | Tinetti Gait | Tinetti Total | GSS | FIM | Rankin Scale |  |
| --- | --- | --- | --- | --- | --- | --- | --- | --- | --- |
| **TUG** |  |  |  |  |  |  |  |  | |
| testDuration | 0.52 | 0.37 | -0.60 | -0.68 | -0.73 | 0.63 | -0.54 | 0.64 | |
| walkTime | 0.51 | 0.36 | -0.62 | -0.70 | -0.75 | 0.66 | -0.51 | 0.63 | |
| standTime |  |  |  |  |  |  |  | 0.32 | |
| sitTime | 0.33 | 0.34 | -0.44 | -0.49 | -0.52 | 0.41 | -0.51 | 0.48 | |
| totalSteps | 0.49 |  | -0.56 | -0.75 | -0.74 | 0.66 | -0.45 | 0.59 | |
| cadence |  |  |  |  |  |  |  |  | |
| strideLength | -0.51 |  | 0.55 | 0.77 | 0.75 | -0.68 | 0.44 | -0.62 | |
| doubleSupport | 0.44 | 0.35 | -0.55 | -0.75 | -0.75 | 0.72 | -0.46 | 0.64 | |
| gaitSpeed | -0.52 | -0.33 | 0.59 | 0.73 | 0.75 | -0.69 | 0.46 | -0.65 | |
| trunkInclination |  |  |  | -0.44 | -0.36 |  | -0.36 | 0.36 | |
| maxTC1 |  |  | 0.41 | 0.58 | 0.56 | -0.53 |  | -0.46 | |
| maxTC2 | -0.35 |  | 0.43 | 0.64 | 0.61 | -0.58 |  | -0.51 | |
| minTC |  |  | 0.48 | 0.57 | 0.58 | -0.52 |  | -0.42 | |
| pitchAtTC2 |  |  | 0.33 | 0.43 | 0.42 | -0.32 |  |  | |
| pci |  |  |  | -0.43 | -0.38 | 0.35 |  |  | |
| strideSD |  |  |  | -0.49 | -0.40 |  |  | 0.38 | |
| psdF |  |  |  |  |  |  |  |  | |
| psdW | -0.37 |  |  |  |  |  |  | -0.44 | |
| **18mW** |  |  |  |  |  |  |  |  | |
| testDuration | 0.58 | 0.39 | -0.56 | -0.72 | -0.75 | 0.68 | -0.55 | 0.69 | |
| totalSteps | 0.56 |  | -0.49 | -0.72 | -0.71 | 0.64 | -0.50 | 0.64 | |
| cadence | -0.34 |  |  |  |  |  |  | -0.32 | |
| strideLength | -0.54 |  | 0.48 | 0.72 | 0.70 | -0.65 | 0.51 | -0.60 | |
| doubleSupport | 0.43 | 0.33 | -0.48 | -0.68 | -0.66 | 0.61 | -0.46 | 0.60 | |
| gaitSpeed | -0.57 | -0.38 | 0.55 | 0.72 | 0.74 | -0.69 | 0.51 | -0.67 | |
| trunkInclination |  |  |  | -0.47 | -0.36 |  | -0.47 | 0.38 | |
| maxTC1 |  |  |  | 0.38 | 0.36 | -0.36 |  |  | |
| maxTC2 | -0.36 |  | 0.34 | 0.53 | 0.53 | -0.53 |  | -0.39 | |
| minTC |  |  |  | 0.37 | 0.38 | -0.38 |  |  | |
| pitchAtTC2 |  |  |  | 0.37 |  |  |  |  | |
| pci | 0.57 |  | -0.36 | -0.62 | -0.57 | 0.48 |  | 0.38 | |
| strideSD | 0.48 |  | -0.46 | -0.70 | -0.66 | 0.63 | -0.37 | 0.51 | |
| psdF | -0.38 |  | 0.33 |  | 0.37 | -0.38 |  | -0.43 | |
| psdW |  |  |  |  |  |  |  |  | |

**Table S2.** Clinical and instrumental variables results relative to TUG and 18mW. Clinical variables are summarised with their median preTT and postVPS; Δ_postVPS-preTT_ indicates the location parameter estimated by the Wilcoxon-Mann-Whitney test. Instrumental variables are summarised with the mean preTT and postVPS; Δ_postVPS-preTT_ indicates their difference; estimations are made with linear mixed-effect models. Relative reliability (ICC), absolute reliability (SEM), and individual-level responsiveness (MDC) of instrumental variables are also reported. ICC=intraclass correlation coefficient, SEM=standard error of measurement, MDC=minimal detectable change. The variables whose MDC is lower than the **Δ_postVPS-preTT_** (95% CI) are in bold.

|  | **preTT** | **postVPS** | **Δ_postVPS-preTT_ (95% CI)** | **p-value** | **ICC 2,k** | **SEM** | MDC |
| --- | --- | --- | --- | --- | --- | --- | --- |
| ***Clinical variables*** |  |  |  |  |  |  |  |
| nSteps2turn | 5 | 3 | -2 (-3,-1.5) | **1.35E-05** |  |  |  |
| Tinetti Balance | 13 | 15 | 2 (1.5,3) | **3.60E-05** |  |  |  |
| Tinetti Gait | 8 | 10 | 2.5 (1.5,3.5) | **4.31E-05** |  |  |  |
| Tinetti Total | 20 | 24 | 4.5 (3.5,5.5) | **1.22E-05** |  |  |  |
| GSS | 5 | 2 | -2.5 (-3.5,-1.5) | **2.20E-04** |  |  |  |
| iNPH-GS | 6 | 3 | -2.5 (-3,-1.5) | **1.51E-05** |  |  |  |
| FIM | 118.5 | 120 | 2.5 (-4,8.5) | 3.21E-01 |  |  |  |
| Rankin Scale | 2 | 1 | -1 (-1.5,-0.5) | **2.21E-03** |  |  |  |
| ***TUG*** |  |  |  |  |  |  |  |
| **testDuration** (s) | 24.07 | 16.41 | -7.66 (-9.47,-6.09) | **4.50E-14** | 0.98 | 1.79 | 4.96 |
| **totalSteps** (steps) | 24.11 | 18.29 | -5.82 (-6.96,-4.66) | **2.11E-19** | 0.97 | 1.79 | 4.95 |
| **walkTime** (s) | 20.83 | 13.95 | -6.88 (-8.68,-5.08) | **5.07E-14** | 0.99 | 1.21 | 3.36 |
| standTime (s) | 3.21 | 2.39 | -0.82 (-1.32,-0.32) | **5.57E-04** | 0.50 | 1.83 | 5.08 |
| sitTime (s) | 4.28 | 3.48 | -0.79 (-1.14,-0.45) | **6.30E-05** | 0.76 | 1.07 | 2.97 |
| turnSteps (steps) | 4.77 | 3.62 | -1.15(-1.58, -0.69) | **2.71e-08** | 0.89 | 0.98 | 2.72 |
| cadence (steps/min) | 47.3 | 50.74 | 3.44 (2.32,4.58) | **2.27E-09** | 0.97 | 1.39 | 3.85 |
| **strideLength** (cm) | 66.02 | 78.53 | 12.51 (10.02,14.73) | **2.97E-25** | 0.99 | 2.22 | 6.16 |
| **doubleSupport** (% of gait cycle) | 41.17 | 36.56 | -4.6 (-5.38,-3.75) | **4.74E-24** | 0.97 | 1.21 | 3.36 |
| **gaitSpeed** (cm/s) | 51.49 | 65.45 | 13.97 (11.78,16.27) | **8.89E-28** | 0.99 | 2.25 | 6.24 |
| trunkInclination (degrees) | 3.29 | 2.38 | -0.91 (-3.14,0.85) | 3.60E-01 | 0.99 | 1.39 | 3.85 |
| **maxTC1** (cm) | 4.48 | 5.18 | 0.7 (0.49,0.9) | **8.57E-11** | 0.98 | 0.23 | 0.63 |
| **maxTC2** (cm) | 3.65 | 4.64 | 0.98 (0.8,1.21) | **3.51E-21** | 0.96 | 0.24 | 0.66 |
| **minTC** (cm) | 1.66 | 2.32 | 0.66 (0.5,0.86) | **9.26E-20** | 0.92 | 0.22 | 0.60 |
| pitchAtTC2 (degrees) | -9 | -18.49 | -9.49 (-23.99,1.14) | 1.55E-01 | 0.89 | 15.04 | 41.70 |
| pci | 28.13 | 21.42 | -6.72 (-12.82,-0.68) | **3.18E-02** | 0.51 | 23.03 | 63.84 |
| strideSD (centisec) | 6.65 | 5.44 | -1.21 (-1.92,-0.70) | **2.54E-04** | 0.90 | 1.14 | 3.15 |
| psdF (Hz) | 1.57 | 1.66 | 0.09 (0.06,0.13) | **6.00E-08** | 0.97 | 0.04 | 0.12 |
| psdW | 0.9 | 1.19 | 0.29 (0.2,0.36) | **4.11E-15** | 0.91 | 0.11 | 0.29 |
| ***18mW*** |  |  |  |  |  |  |  |
| **testDuration** | 33.85 | 25.76 | -8.1 (-10.62,-5.73) | **2.08E-11** | 0.99 | 1.40 | 3.87 |
| **totalSteps** | 41.85 | 34.89 | -6.96 (-9.38,-4.85) | **2.78E-11** | 0.99 | 1.27 | 3.52 |
| cadence | 50.36 | 52.69 | 2.33 (1.42,3.29) | **1.38E-07** | 0.97 | 1.31 | 3.63 |
| **strideLength** | 85.37 | 98.53 | 13.16 (10.58,16.54) | **7.10E-21** | 0.99 | 1.96 | 5.44 |
| **doubleSupport** | 35.87 | 33.22 | -2.66 (-3.54,-1.95) | **3.61E-09** | 0.99 | 0.65 | 1.80 |
| **gaitSpeed** | 71.8 | 86.99 | 15.19 (12.02,18.87) | **1.01E-24** | 0.99 | 2.26 | 6.25 |
| trunkInclination | -1.2 | -1.24 | -0.04 (-1.11,1.28) | 9.52E-01 | 1.00 | 0.65 | 1.81 |
| maxTC1 | 5.41 | 5.66 | 0.25 (0.03,0.43) | **1.72E-02** | 0.99 | 0.19 | 0.54 |
| **maxTC2** | 4.33 | 5.39 | 1.06 (0.85,1.26) | **1.49E-23** | 0.96 | 0.28 | 0.77 |
| **minTC** | 1.88 | 2.5 | 0.62 (0.51,0.78) | **4.18E-21** | 0.96 | 0.18 | 0.49 |
| pitchAtTC2 | -27.65 | -45.08 | -17.43 (-41.13,10.47) | 1.10E-01 | 0.80 | 37.81 | 104.81 |
| pci | 14.64 | 11.62 | -3.02 (-5.14,-1.44) | **4.64E-04** | 0.80 | 4.47 | 12.40 |
| strideSD | 3.18 | 2.73 | -0.45 (-0.68,-0.19) | **1.21E-04** | 0.94 | 0.34 | 0.94 |
| **psdF** | 1.63 | 1.75 | 0.12 (0.09,0.15) | **5.28E-13** | 0.98 | 0.03 | 0.09 |
| psdW | 0.7 | 0.83 | 0.13 (0.06,0.2) | **3.07E-04** | 0.87 | 0.13 | 0.35 |
